# Validated method for phytohormone quantification in plants

**DOI:** 10.3389/fpls.2014.00417

**Published:** 2014-08-26

**Authors:** Marília Almeida Trapp, Gezimar D. De Souza, Edson Rodrigues-Filho, William Boland, Axel Mithöfer

**Affiliations:** ^1^Department Bioorganic Chemistry, Max Planck Institute for Chemical EcologyJena, Germany; ^2^LaBioMMi, Chemistry Department, Federal University of São CarlosSão Carlos, Brazil; ^3^Accert Chemistry and Biotechnology Inc.São Carlos, Brazil

**Keywords:** phytohormones, HPLC-MS/MS, quantification, *Arabidopsis thaliana*, *Citrus sinensis*, iontrap

## Abstract

Phytohormones are long time known as important components of signaling cascades in plant development and plant responses to various abiotic and biotic challenges. Quantifications of phytohormone levels in plants are typically carried out using GC or LC-MS/MS systems, due to their high sensitivity, specificity, and the fact that not much sample preparation is needed. However, mass spectrometer-based analyses are often affected by the particular sample type (different matrices), extraction procedure, and experimental setups, i.e., the chromatographic separation system and/or mass spectrometer analyser (Triple-quadrupole, Iontrap, TOF, Orbitrap). For these reasons, a validated method is required in order to enable comparison of data that are generated in different laboratories, under different experimental set-ups, and in different matrices. So far, many phytohormone quantification studies were done using either QTRAP or Triple-quadrupole mass spectrometers. None of them was performed under the regime of a fully-validated method. Therefore, we developed and established such validated method for quantification of stress-related phytohormones such as jasmonates, abscisic acid, salicylic acid, IAA, in the model plant *Arabidopsis thaliana* and the fruit crop *Citrus sinensis*, using an Iontrap mass spectrometer. All parameters recommended by FDA (US Food and Drug Administration) or EMEA (European Medicines Evaluation Agency) for validation of analytical methods were evaluated: sensitivity, selectivity, repeatability and reproducibility (accuracy and precision).

## Introduction

Phytohormones constitute a distinct class of signaling molecules in plants. They can be classified according to their chemical structure—jasmonates [jasmonic acid (JA) and derivatives 12-oxo-phytodienoic acid (OPDA)], auxins (in particular indole-3-acetic acid, IAA), cytokinins, gibberellins, abscisic acid (ABA), salicylic acid (SA), brassinosteroids, ethylene—or according to their biological function—regulator of plant growth, development and reproduction or mediators during biotic and abiotic stresses (Santner and Estelle, 2009).

Frequently these molecules act at low concentrations and play key roles in ecological interactions between plants and other organisms (Pozo et al., 2005; Pieterse et al., 2009; Santner et al., 2009) Therefore, quantification of phytohormones is an essential step to understand their functions in plant metabolism and ecological interactions. While the first highly sensitive methods for quantitative phytohormone analyses relied on immunoassays (Weiler, 1984), in the last 15–20 years many methods have been developed for quantification of these compounds, particularly using hyphenated techniques such as GC-MS (Kowalczyk and Sandberg, 2001; Müller et al., 2002, 2006; Engelberth et al., 2003) and LC-MS (Wilbert et al., 1998; Forcat et al., 2008; Pan et al., 2008, 2010; Müller and Munné-Bosch, 2011; Balcke et al., 2012; Liu et al., 2012).

These techniques provide a powerful analytical tool for quantifying secondary metabolites in plant tissues, especially due to their high sensitivity, specificity and reproducibility. However, different approaches might be adopted depending on the separation method (GC or HPLC) and the spectrometer (triple quadrupole, iontrap, TOF) applied during the quantification studies. Moreover, mass spectrometry analyses are strongly influenced by other compounds present in the plant tissues which can suppress or increase the analyte ionization, a fact that is often not considered. Hence, the matrix effect and several other parameters (like analyte stability and recovery) must be controlled during a quantification study and validation strategies should be employed in order to produce reliable analytical methods for quantification of plant metabolites.

Several papers and reviews covering validation of analytical methods have been published (Shabir, 2003; Bliesner, 2006; Chandran and Singh, 2007). As a rule, these papers describe important parameters such as accuracy, precision (repeatability and intermediate precision), specificity, detection and quantification limits, linearity, range, robustness, etc. All this set of information should be obtained in the same laboratory as a part of repeatability assays. However, for proceeding with reproducibly assays an inter-laboratory experiment is often necessary. Collaborative trials are used to test the performance (generally the precision) of the analytical method demonstrating that it can be used in more than one laboratory, producing reliable and true results (Hund et al., 2000).

In this present paper we describe the development and inter-laboratory validation of an analytical method for quantification of six phytohormones—the auxin indole-3-acetic acid (IAA), ABA, JA, isoleucine jasmonic acid conjugate (JA-Ile), SA, and 12-oxo phytodienoic acid (OPDA)- in *Arabidopsis thaliana* and *Citrus sinensis* using an iontrap mass spectrometer.

## Materials and methods

### Reagents and standards

All solvents used during extraction procedures were analytical grade except for methanol (MeOH). Chromatographic separation was carried out using MeOH HPLC grade purchased from Roth (Carl Roth GmbH, Germany) or J. T. Baker (Xalostoc, Mexico). IAA (purity > 99%), ABA (purity > 99%) and SA (>98%) were purchased from Sigma-Aldrich. 12-oxo phytodienoic acid were purchased from Cayman (Biomol GmbH, Hamburg, Germany). JA was synthesized by saponification of commercially available methyl-JA. Jasmonic acid isoleucine conjugate (JA-Ile) was synthetized according to Kramell et al. (1988). Deuterated standards: [^2^H5] indole-3-acetic acid (d5-IAA), [^2^H4] salicylic acid (d4-SA) and [^2^H6] (+)-cis, trans-abscisic acid (d6-ABA) were purchased from OlChemIm Ltd (Olomouc, Czech Republic) and jasmonic-d5 acid 2,4,4-d3 acetyl-2,2-d2 (d5-JA) was purchased from CDN isotopes (Quebec, QC, Canada).

### Apparatus

HPLC-MS/MS analysis was performed on an Agilent 1100 HPLC system (Agilent Technologies, Böblingen, Germany) connected to a LTQ Orbitrap mass spectrometer (Thermo Scientific, Bremen, Germany). Chromatographic separation was carried out in a Luna Phenyl-Hexyl column (150 × 4.6 mm, 5 μm; Phenomenex, Aschaffenburg, Germany). Formic acid (0.05%, v/v) and MeOH with 0.05% (v/v) of formic acid were employed as mobile phases A and B, respectively. The elution profile was: 0–10 min, 42–55% B in A; 10–13 min, 55–100% B; 13–15 min 100% B; 15–15.1 min 100–42% B in A; and 15.1–20 min 42% B in A. The mobile phase flow rate was 1.1 mL/min. Injection volume was 25 μL. The LTQ mass spectrometer was equipped with an Electrospray ionization source, operating in the negative and positive ion modes. Negative measurements were carried out using the following ionization parameters: source voltage: 4.4 kV, capillary voltage: −48 V, tube lens −113 V, declustering potential 10 V, turbo gas temperature: 300°C, auxiliary gas flow: 4.5 L/min, sheath gas flow: 9 L/min. For positive analyses ionization parameters were set at: source voltage: 4.2 kV, capillary voltage: 29 V, tube lens 45 V, declustering potential 10 V, turbo gas temperature: 300°C, auxiliary gas flow: 4.5 L/min, sheath gas flow: 9 L/min.

Selected reaction monitoring (SRM) experiments were used to monitor specific precursor ion → product ion transitions for each phytohormone and internal standard. Collision energy, precursor ion isolation width and activation Q were optimized for each compound separately.

During the inter-laboratory reproducibility, the analyses were performed in an Acquity HPLC (Waters Co.) coupled with Quattro Premier XE (Micromass Technology) mass spectrometer, using a Luna Phenyl-Hexyl column (150 × 4.6 mm, 5 μm; Phenomenex, Aschaffenburg, Germany) and the same elution conditions mentioned above. The ionization parameters used during these analyses were: In negative mode (capillary: 3.4 kV, extractor 3 V, source temperature 110°C, desolvation temperature 350°C, desolvation gas flow: 800 L/h, cone gas flow: 10 L/h), and in positive mode (capillary: 3.4 kV, extractor 3V, source temperature 110°C, desolvation temperature 350°C, desolvation gas flow: 800 L/h, cone gas flow: 10 L/h). Cone voltage and collision energy were optimized for each compound individually.

### Plant material

*A. thaliana* was cultured for 4 weeks under short day conditions (10 h light/14 h dark photoperiod), 40% humidity and 23°C. After harvesting, plants were immediately frozen in liquid nitrogen and ground in a GenoGrinder (SPEXSample Prep, München, Germany) for 2 × 30 s at 1500 rpm. After homogenization, 100 mg of plants were weighted into 1.5 mL tubes and stored at −80°C until the measurements.

*C. sinensis* was cultured in a greenhouse (Araraquara, Brazil) under normal light conditions and temperature average of 26°C (day) and 18°C (night). Light green leaves from small trees were collected, immediately frozen in liquid nitrogen and ground in a mortar. After homogenization, 100 mg of frozen plant material were weighted into 1.5 mL tubes and stored at −80°C until the measurements.

### Phytohormones extraction and analysis

#### Optimization of phytohormones extraction

Two parameters were evaluated during the optimization of phytohormones extraction: composition of extraction solution and type of plant samples (fresh or dry material). Initially tubes containing 100 ± 1 mg of plant material were either kept at −80°C or dried overnight in a freeze drier at −42°C. The extraction was performed adding 1.0 mL of either ethyl acetate, dichloromethane, isopropanol, MeOH or MeOH:water (8:2) into each tube containing dry or fresh plant material. Samples were shaken for 30 min in the Starlab shaker and centrifuged at 16,000 g and 4°C for 5 min. The supernatant was transferred into a new 1,5 micro-centrifuge tube and dried in speed vac. After drying, 100 μL of MeOH were added to each sample, homogenized under vortex and centrifuged at 16,000 g and 4°C for 10 min. The supernatant was analyzed by HPLC-MS/MS.

In a second set of analyses, the influence of both MeOH:water ratio and addition of acid in the extraction mixture was evaluated. The extraction procedure was performed as described above using 3 different MeOH:water ratios (7:3, 6:4, and 1:1) pure, or containing 0.2% of HCl.

#### Preparation of standards solutions

Stock solutions of each original phytohormone standard were prepared at 1 mg/mL in MeOH. For deuterated compounds, stock solutions were prepared in acetonitrile at 100 μg/mL.

Working solutions of original phytohormones standards were prepared diluting stock solutions in MeOH:water (7:3), at different concentration for each phytohormone depending on the range of the calibration curve: ABA and IAA (100 μg/mL), JA and SA (200 μg/mL), OPDA (50 μg/mL), and JA-Ile (40 μg/mL).

The internal standard stock solutions (d5-JA, d6-ABA, d4-SA, and d5-IAA) were combined and diluted (final concentration 10 ng/mL for d4-SA and d5-IAA and 20 ng/mL for d5-JA and d6-ABA) with MeOH:water (7:3) yielding the extraction solution.

#### Final method for phytohormones extraction

Tubes containing 100 mg of fresh and ground plant material were kept at −80°C, and transferred to liquid nitrogen before the extraction. The samples were removed from the liquid nitrogen and 1 mL of extraction solution containing the internal standards (d5-JA, d6-ABA, d5-IAA, and d4-SA), prepared as described in Preparation of Standards Solutions, were directly added. The samples were briefly mix with a vortex, and spiked with phytohormones standards as described in Method Validation to generate the calibration curve and quality control (QC) samples. The spiked samples were shaken for 30 min in the Starlab shaker and centrifuged at 16,000 g and 4°C for 5 min. The supernatant was transferred into a new 1,5 micro-centrifuge tube and dried in speed vac. After drying, 100 μL of MeOH were added to each sample, vortexed and centrifuged at 16,000 g and 4°C for 10 min. The supernatant was analyzed by HPLC-MS/MS.

### Method validation

#### Limit of detection and limit of quantification

The limits of detection (LOD) and quantification (LOQ) for analytical methods based on HPLC analysis can be expressed in response units (signal-to-noise levels). Usually LOD is established using matrix samples spiked with the low amount of standards. However, as none analyte-free matrix was available the LODs were determined in solvent as three times the noise level.

For each matrix, LOQs were defined according to the amount of phytohormones present in 10 independent blank samples, which were extracted as described in Optimization of Phytohormones Extraction. For all the LOQ the signal-to-noise ratios were higher than 10.

#### Calibration curve and linearity

The calibration curves were prepared in matrix using three different spiking solutions: spiking solution A containing ABA (at 4, 8, 40, 100, 200, 1000, 3000, and 4000 ng/mL), IAA (2, 4, 20, 50, 100, 500, 1000, 2000 ng/mL), and JA-Ile (0.8, 1.6, 8, 20, 40, 200, 400, and 800 ng/mL); spiking solution B containing SA (at 50, 100, 200, 500, 1000, 2000, 4000, and 8000 ng/mL) and JA (at 25, 50, 100, 250, 500, 1000, 2000, and 4000 ng/mL); and spiking solution C containing OPDA (at 500, 1000, 2000, 4000, 6000, 7000, 8000, and 10,000 ng/mL). All spiking solutions were prepared (in MeOH:water, 7:3) by serial dilution of working solutions.

Samples for calibration curve were prepared adding 50 μL of each spiking solution (A, B, and C) into the tubes containing 100 mg of ground fresh plant material and extracted as described in Preparation of Standards Solutions. For a flow sheet see Scheme 1 (Supporting Material).

#### Quality controls

QC were used to assess the method's accuracy and precision. QCs were prepared spiking plant material with three different levels of each phytohormone (low, medium and high; Scheme 1, Supporting Material).

High quality controls (HQC) were prepared spiking 100 mg of plant material with 50 μL of: high spiking solution A (containing 2800 ng/mL of ABA and IAA and 280 ng/mL of JA-Ile); high spiking solution B (containing 5600 ng/mL of SA and 2800 ng/mL JA) and high spiking solution C (containing 2800 ng/mL of OPDA). Medium quality controls (MQC) were prepared spiking 100 mg of plant material with 50 μL of: medium spiking solution A (containing 700 ng/mL of ABA and IAA and 140 ng/mL of JA-Ile); medium spiking solution B (containing 2800 ng/mL of SA and 1400 ng/mL of JA) and medium spiking solution C (containing 1400 ng/mL of OPDA). And low quality controls (LQC) were prepared spiking 100 mg of plant material with 50 μL of: low spiking solution A (containing 14 ng/mL of ABA and IAA, and 2.8 ng/mL of JA-Ile); low spiking solution B (containing 280 ng/mL of SA and 140 ng/mL of JA) and low spiking solution C (containing 450 ng/mL of OPDA). All QC were prepared in quintuplicates.

#### Recovery

Recovery was calculated comparing the amount of each phytohormone present in spiked/extracted and extracted/spiked QC. The spiked/extracted QC were prepared as described in Quality Controls. The extracted/spiked samples were spiked with 150 μL of MeOH:water (7:3)—simulating the addition of spiking solutions—and extracted as described in Preparation of Standards Solutions. The dry residues were reconstituted in MeOH containing the final concentration of each phytohormone, which corresponds to half of spiking solution concentration.

#### Validation in Citrus sinensis

Linearity, reproducibility, recovery, and matrix effects were also evaluated for quantification of phytohormones in leaves of orange, *C. sinensis*. Initially, 10 samples were analyzed to establish the basal level of the six phytohormones in *C. sinensis* tissues. Due to the high content of IAA and ABA and low content of OPDA, the range of calibration curves and QC levels were adjusted to better fit to the new matrix.

The calibration curves were prepared in matrix using three different spiking solutions: spiking solution A contained ABA (at 4, 8, 40, 100, 200, 1000, 3000, and 4000 ng/mL), and JA-Ile (0.8, 1.6, 8, 20, 40, 200, 400, and 800 ng/mL); spiking solution B containing SA (at 50, 100, 200, 500, 1000, 2000, 4000, and 8000 ng/mL), JA (at 25, 50, 100, 250, 500, 1000, 2000, and 4000 ng/mL) and IAA(25, 50, 100, 250, 500, 1000, 2000, and 4000 ng/mL); and spiking solution C contained OPDA (at 60, 120, 240, 480, 640, 800, 1000, and 1200 ng/mL). All spiking solutions were prepared (in MeOH:water, 7:3) by serial dilution of working solutions. Samples for calibration curves were prepared adding 50 μL of each spiking solution (A, B, and C) into the tubes containing 100 mg of ground fresh plant material and extracted as described in Preparation of Standards Solutions. For a flow sheet see Scheme 1 (Supporting Material).

HQC were prepared by spiking 100 mg of plant material with 50 μL of: high spiking solution A (containing 2800 ng/mL of ABA and 280 ng/mL of JA-Ile); high spiking solution B (containing 5600 ng/mL of SA and 2800 ng/mL JA and IAA) and high spiking solution C (containing 840 ng/mL of OPDA). MQC were prepared spiking 100 mg of plant material with 50 μL of: medium spiking solution A (containing 700 ng/mL of ABA and 140 ng/mL of JA-Ile); medium spiking solution B (containing 2800 ng/mL of SA and 1400 ng/mL of JA and IAA) and medium spiking solution C (containing 600 ng/mL of OPDA). And LQC were prepared spiking 100 mg of plant material with 50 μL of: low spiking solution A (containing 14 ng/mL of ABA and 2.8 ng/mL of JA-Ile); low spiking solution B (containing 280 ng/mL of SA and 140 ng/mL of JA and IAA) and low spiking solution C (containing 90 ng/mL of OPDA). All QC were prepared in quintuplicates.

Recovery of phytohormones in *C. sinensis* samples was evaluated for the QC samples as described in Recovery.

## Results and discussion

### Method development

#### Optimization of ion trap parameters for quantification of phytohormones

Due to their high sensitivity, specificity, and the fact that not much sample preparation is necessary, HPLC-MS/MS experiments, especially those involving SRM, are used as reference for quantitative analyses. These also include phytohormone quantifications.

SRM experiments are based on two stages of ion selection. The precursor ion (a protonated or deprotonated target molecule) is selected in the first stage of tandem mass spectrometer, fragmented under a controlled process, thereby generating a specific fragment ion, which is then selected in the second stage of tandem mass spectrometer. Hence, the specificity of SRM experiments relies upon the choice of a specific precursor-fragment ion transition, while the sensitivity depends on the yield and stability of both precursor and fragment ions (Kowalczyk and Sandberg, 2001). Moreover, selection of precursor and fragment ions as well as fragmentation mechanism occurs in different ways for distinct mass spectrometers (triple quadrupole, ion trap, time of flight). Therefore, different approaches and parameters optimization are needed depending on which kind of detector is used in the SRM experiments.

Quantification of phytohormones in plant tissues has been so far carried out using either triple quadrupole or Q-trap instruments (Forcat et al., 2008; Pan et al., 2008, 2010; Balcke et al., 2012; Liu et al., 2012), which are well known for their high performance in SRM experiments (Rousu et al., 2010; Tanaka et al., 2011) Ion trap mass spectrometers, on the other hand, are widely available due to their high versatility, capability of doing MS^n^, and for its low cost compared with triple quadrupole, which make it an attractive option for compound identification, screening and qualitative analyses. However, they present specific challenges for quantification experiments, since the scan speed and fragmentation mode do not fit the best with SRM experiments. Therefore, many parameters must be carefully adjusted in order to reach good sensitivity in ion trap mass spectrometers, specially the injection time and activation Q (Evans et al., 2000).

During the present work all parameters for ionization, fragmentation and detection of phytohormones (ABA, IAA, SA, JA, JA-Ile, and OPDA) were optimized, in order to achieve good sensitivity and selectivity in an ion trap mass spectrometer. The values of precursor ion isolation width (ISO), collision energy (CID) and activation Q (Act Q) that presented the best sensitivity and the more stable signals for each phytohormone are shown in Table 1. Activation Q must be adjusted before choosing the product ion, since it determines the range of product ions that can be generated. Modification in the default value (0.250 for the equipment used in this work) can provide new fragment ions, which can be interesting for quantification (stable and with high intensity). Injection time for all SRM transitions was 100 ms.

**Table 1:** **Fragmentation parameters for the phytohormones**.

	**Precursor ion (m/z)**	ISO^*^ (Da)	CID^**^ (V)	Act Q^***^	**Fragments (Da)**
ABA	263.0	2.0	30	0.250	152.0–154.0
d6-ABA	269.0	2.0	30	0.250	158.0–160.0
IAA	176.0	2.0	20	0.250	129.0–131.0
d5-IAA	181.0	2.0	20	0.250	134.0–136.0
JA	209.0	1.0	25	0.210	58.0–60.0
d5-JA	214.0	1.0	25	0.210	61.0–63.0
JA Ile	322.0	2.0	30	0.250	129.0–131.0
OPDA	291.0	2.0	18	0.250	164.0–166.0
SA	137.0	1.0	28	0.250	92.0–94.0
d4-SA	141.0	2.0	28	0.250	96.0–98.0

* Precursor ion isolation window;

** Collision-induced dissociation energy;

*** Activation Q.

#### Optimization of phytohormones extraction

The efficiency of phytohormones' extraction was evaluated for both dry and fresh plant material using different organic solvents/mixtures [acetate, dichloromethane, isopropanol, MeOH, MeOH:water (8:2), MeOH:water (7:3), MeOH:water (6:4), MeOH:water (1:1)]. The influence of acidification by hydrogen chloride in the phytohormone extraction was also tested. The results are presented in Figure A (Supplementary Material). When the extraction is performed using non-polar organic solvents (ethyl acetate and dichloromethane) there is a clear difference in the extraction efficiency between fresh and dry material. However, for polar and aqueous mixtures such difference decreased drastically. Mixtures of MeOH and water provided higher extraction efficiency for all phytohormones. Here, the ratio of 7:3 was chosen as extraction solution due to its good performance in extracting the phytohormones and the low content of chlorophyll present in the final sample.

**Figure 1:**
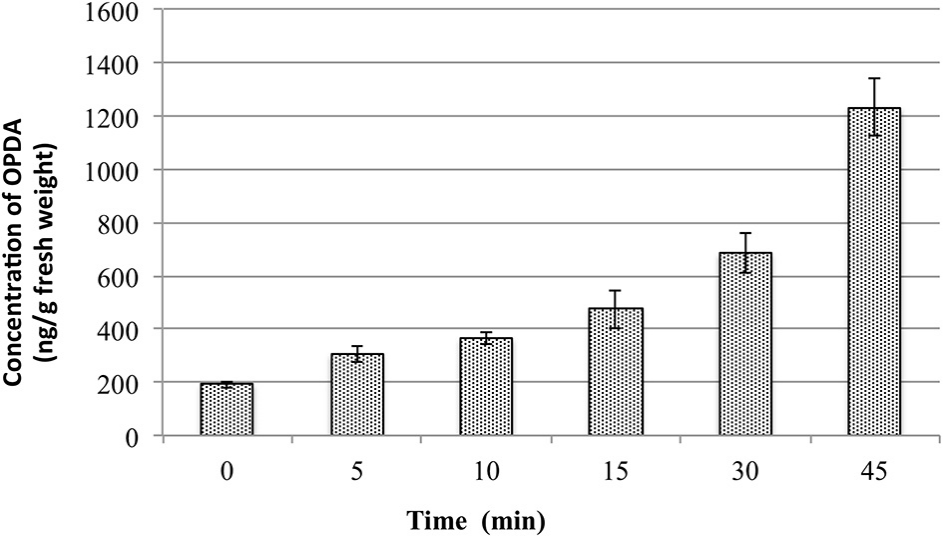
**Changes in OPDA content in *Arabidopsis thaliana* leaf samples depending on the time the samples were kept on ice**.

During the evaluation of method repeatability, the concentration of OPDA in the QC samples did not fit with the added amount. The concentration present in the QC was always higher than expected. After more detailed analyses it was observed that such issue occurred due to the increase of OPDA content in the plant samples during the sample preparation. Actually, all the samples were put on ice, spiked with internal standards and extracted by addition of extraction solution containing the internal standards. As the QC were prepared after the calibration curve samples, the increment in OPDA content in the QC was bigger than in the calibration curve samples. Therefore, the changes in the OPDA content in the plants samples were evaluated while QCs were kept on ice. For this purpose, 18 tubes containing 100 mg of fresh and ground plant tissues were transferred from liquid nitrogen onto ice. The OPDA concentration was evaluated for samples kept on ice for 0, 5, 10, 15, 30, and 45 min. For each point, three tubes containing plant material were removed from ice and added with 1.0 mL of extraction solution. The extraction was carried out as described in Final Method for Phytohormones Extraction. The graphs present in Figure 1 shows the changes in OPDA content.

These data suggest that OPDA content varies quickly in the wounded/ground plant tissues even when the samples are kept on ice. After 5 min it increased by 50% and the amount doubled within the first 10 min. This might be due to remaining enzyme activities releasing lipid-bound OPDA from plastids localized galactolipids, which are well known for Arabidopsis (Stelmach et al., 2001). These results showed clearly the importance of keeping plant tissues frozen as long as possible, even during weighting and before adding the extraction solvents. Therefore, samples must be maintained at very low temperature (−80°C or liquid nitrogen) before the extraction, and the extraction solvent must be added immediately after removing the samples from such conditions.

These results also highlight the importance of the validation studies for quantification methods, since many parameters involved in the extraction and analysis cannot be proper addressed when statistical figures are not evaluated. In this way, the use of QC as defined in validation protocols can be of great value even during method development. For this reason, validation of each assay or test method should be performed on a case-by-case basis, to ensure that the parameters are appropriate for the method's intended use.

### Validation of analytical method

The validation studies are conducted in order to demonstrate that the analytical method is applicable for the aimed purpose and to ensure that the obtained values are close to the unknown content of the analyte present in real samples (EMEA, 2006; González and Herrador, 2007; European Commission, 2010). In this work, we evaluated the selectivity/specificity, limits of detection and quantification, linearity, recovery, repeatability and reproducibility of analytical method for quantification of phytohormones in *A. thaliana* and *C. sinensis* tissues.

#### Selectivity, limit of detection and quantification

Selectivity is defined as the ability of quantification method to discriminate the analyte from the other sample components, giving pure, symmetric and resolved peaks (Green, 1996). For methods which include chromatographic separation, selectivity can be assessed by chromatographic resolution, evaluating whether the peak relative to the analyte is separated from the other peaks present in the matrix. When no blank matrix is available, the selectivity can also be assessed comparing the MS/MS spectrum related to the analyte present in the matrix with the MS/MS spectrum of original standard. If there is no additional peaks MS/MS spectrum for the band correspondent to the analyte in the matrix comparing to MS/MS spectrum of original standards, it suggests that the method is selective.

**Figure 2:**
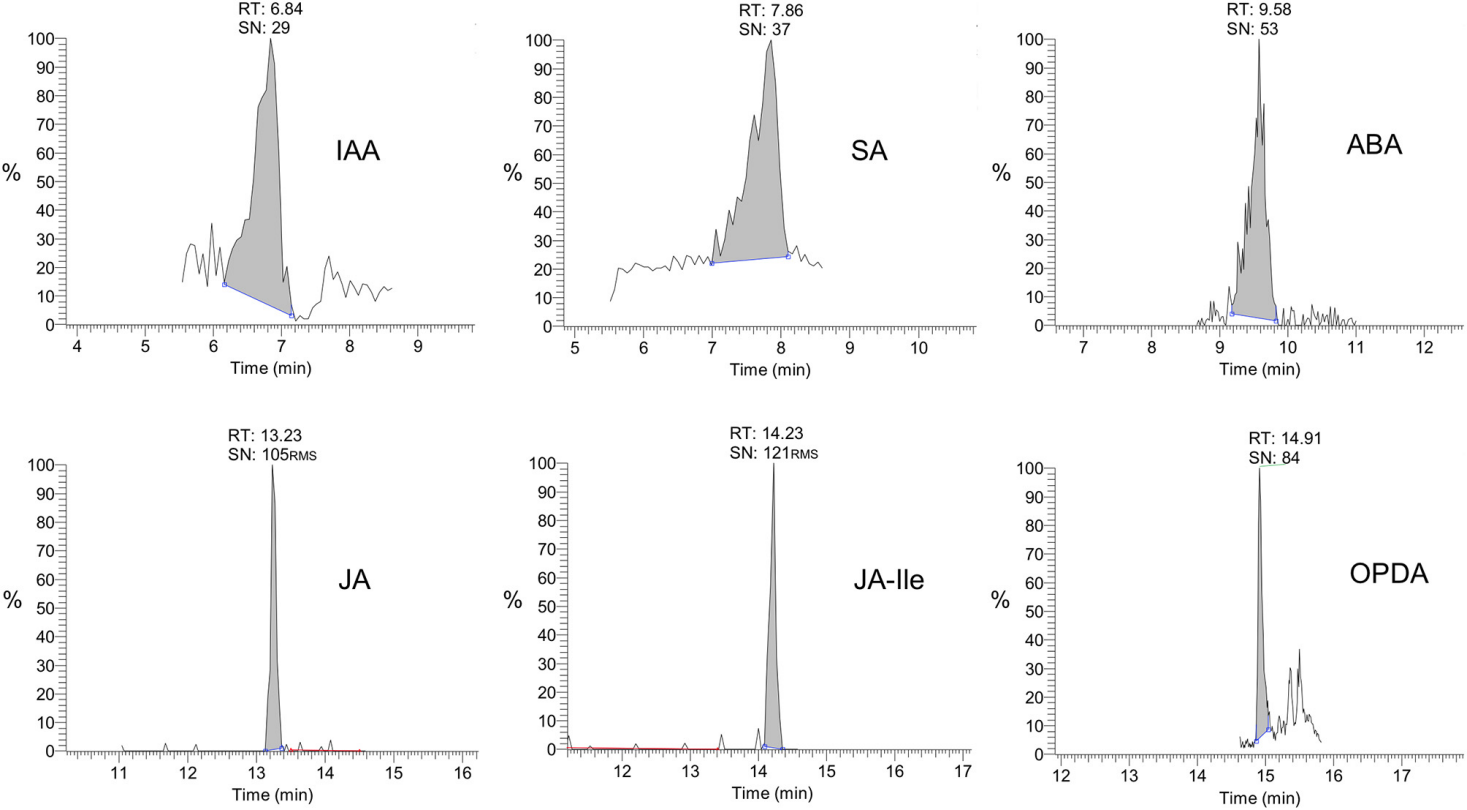
**Selected reaction monitoring (SRM) chromatograms for various phytohormones in *Arabidopsis thaliana* leaf extracts**.

Therefore, the present method is considered selective/specific for the phytohormones quantification, since the SRM chromatograms present in Figure B (Supplementary Material) contain either only one or well-resolved peaks for all phytohormones. For JA, JA-Ile, and OPDA the peaks are very symmetric and sharp (width less than 30 s). Although for IAA and SA the peaks are broader and not symmetric, the selectivity of the method was also confirmed by the very similar profile of MS/MS spectra related to these bands (Figure 2) and the original standards prepared in solvent (Figure B of Supplementary Material).

The limit of detection (LOD) is the lowest analyte concentration, which can be distinguished from the noise in blank samples [it is defined as a concentration with signal/noise (S/N) of 3]. When no analyte-free matrix is available, the detection limit can be calculated in solvent (LOD of the equipment) or by dilution of matrix until reaching an S/N of 3. Since the dilution of the matrix also reduces the matrix effect, thereby not presenting huge advantages compared with the measurements in solvent, in the present work the LODs were evaluated for the HPLC-MS/MS system and the values are shown in Table 2.

**Table 2:** **Parameters of calibration curve for each phytohormone: curve range, regression, weighting, correlation coefficient limit of quantification (LOQ) and amount of each phytohormone present in blank (untreated) *Arabidopsis thaliana* samples**.

**Analyte**	**Range (ng/g FW)**	Curve^*^	**R^2^**	**LOQ (ng/g)**	Amount in blank samples^**^ (ng/g FW)	Matrix effect^***^
IAA	2–2000	Y = 0.0929916 + 0.0239565^*^X	0.992	2.0	7.13 ± 1.62	−31%
ABA	2–2000	Y = 0.0726676 + 0.0159863^*^X	0.998	2.0	5.32 ± 0.88	+11%
JA-Ile	0.4–400	Y = 0.146972 + 0.106572^*^X	0.993	0.4	1.64 ± 0.23	−25%
JA	12.5–2000	Y = 0.335095 + 0.00835023^*^X	0.997	12.5	41.32 ± 7.80	+7%
SA	25–4000	Y = 0.80327 + 0.00608683^*^X	0.989	25.0	123.59 ± 12.89	+46
OPDA	75–2000	Y = 3.03745 + 0.00598094^*^X	0.998	75.0	447.41 ± 57.21	−87%

* A weighting factor of 1/x^2^ was applied to all curves, except for OPDA, which used a factor of 1/x.

** Values are average ± standard deviation. Concentrations represent the amount of each phytohormone in plant tissues (ng/g of fresh weight, FW), which is corresponding to the concentration (ng/mL) in the injection solution.

*** Values correspond to ((m_matrix_/m_solvent_) −1)^*^100%.

The limit of quantification (LOQ) is defined as the lowest analyte concentration, which can be quantified precisely and accurately. According to EMA and FDA it corresponds to the concentration of analyte, which yield a peak with S/N of 10. However, as can be seen in Figure 2, the amount of every phytohormone in the blank sample yield peaks with S/N of at least 30. Therefore, it is not possible to calculate the LOQ using the conventional definition. For this reason the LOQ for this method was established as the lowest point of the calibration curve (Table 2). The SRM chromatogram of this point for every phytohormone is shown in the Figure C (Supplementary Material).

The S/N of the first calibration point for all phytohormones is much higher than 10, which is established as the minimum S/N ration for the LOQ. It proves the lowest calibration limit for all phytohormones is above to the LOQ of this method.

#### Calibration curve and linearity

The range of calibration curves was defined for each compound based on the amount of each compound present in the matrix (Table 2) and the changes that might occur during experiments. It is important that the calibration curves include the concentration of the phytohormones present in the blank (untreated control) samples, since it usually corresponds to the control in biological experiments. Hence, the analytical method must be suitable to quantify the amount of each phytohormone in control samples. Here it should be mentioned that the phytohormone concentrations measured in this study are in the same range as published by other groups (e.g., Müller et al., 2002; Pan et al., 2008).

Both correlation coefficient (*R*^2^) and residual plots were used to evaluate the linearity of calibration curve for each phytohormone.

**Figure 3:**
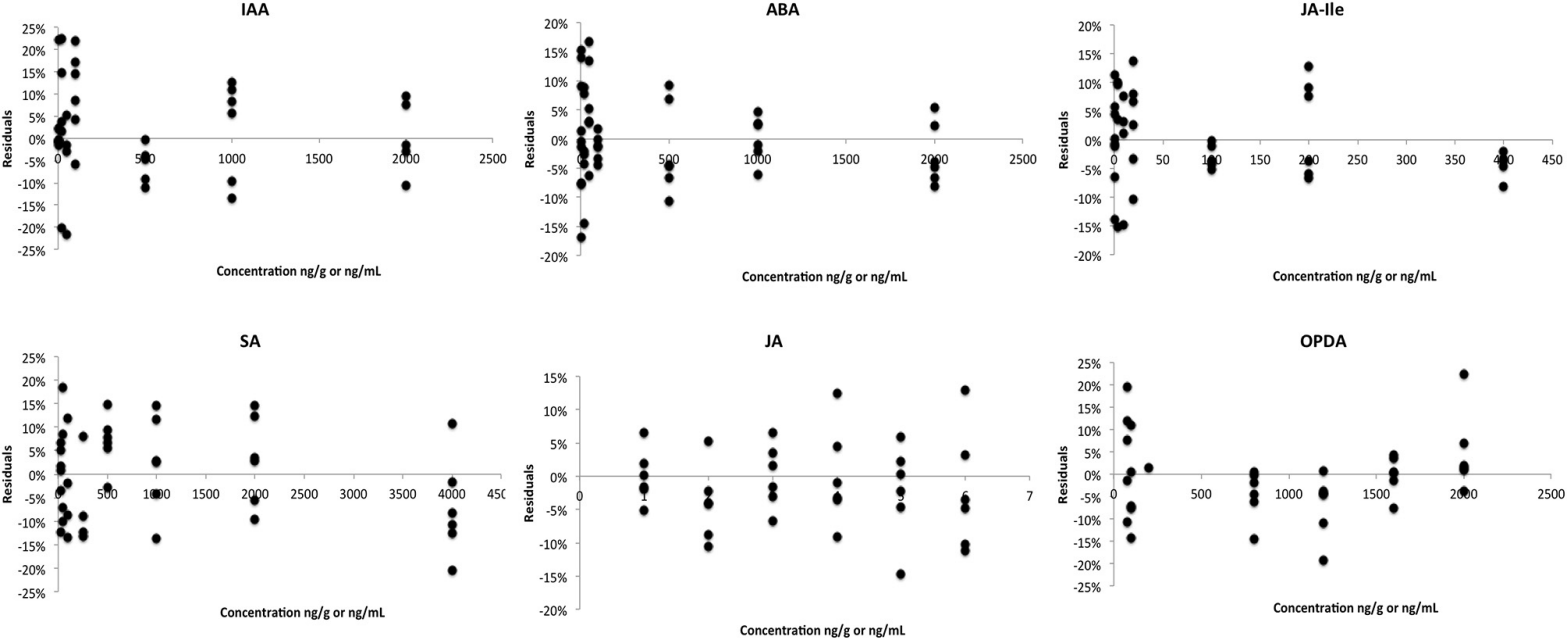
**Residual plot associated with the best regression and weighting for calibration curve of each phytohormone**.

Homoscedasticity tests were performed in order to select the best weighting for the linear regression. In these tests, the residual of each point of calibration curve (difference between the calculated and theoretic values) is plotted against the concentration level. For an adequate regression model (regression and weighting) the residuals are normally distributed along the X-axis (Almeida et al., 2002). To support the data shown in Table 2, Figure 3 presents the residual plots for the best regression and weighting applied to the calibration curve of each phytohormone. For IAA, ABA, JA-Ile, JA, and SA the weighting factor that fits the best to the linear curve is 1/x^2^. For OPDA, it was 1/x. A linear regression was used in the calibration curve for all phytohormones. Thus, those factors and regression were applied in every analytical curve during the whole validation study.

### Matrix effect

The matrix components can affect the analyte stability, extraction and ionization. As was shown above for OPDA, some enzymes present in wounded *A. thaliana* tissues can modify the basal concentration of OPDA, even when the tissues are kept at low temperature (4°C). In other cases, some enzymes can also degrade the analyte or modify the efficiency of analyte extraction. Moreover, for HPLC-MS/MS methods, some constituents of the matrix affect the efficiency of the analyte ionization, when both have the same retention time. In this case, the matrix interferes can either suppress the analyte ionization (decreasing the response) or enhance it (producing higher responses). The effects of matrix on quantitative methods are not completely understood and varies depending on both analyte and matrix composition.

**Figure 4:**
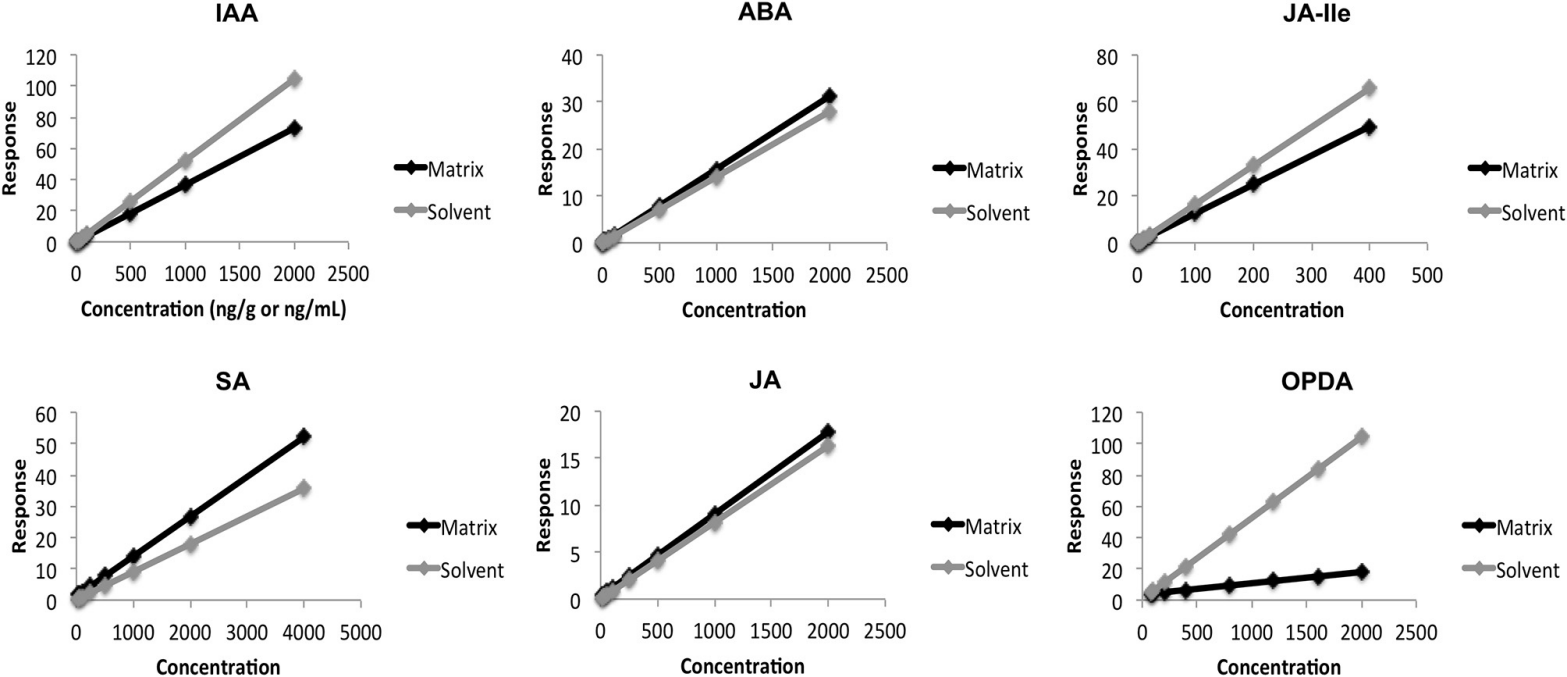
**Comparison between calibration curves performed in the matrix (*Arabidopsis thaliana*) and in solvent**.

During the validation, we evaluated the influence of *A. thaliana* constituents on quantification of every phytohormone, analysing the slope (m) of each calibration curve prepared in both solvent and matrix. Comparison between these slopes (m_matrix_/m_solvent_) showed that the matrix has small influence for quantification of JA and ABA, +7% and +11%, respectively (Table 2 and Figure 4). For the other phytohormones components present in *A. thaliana* affected the measurements in two different ways: increasing the response for SA (+46%) and decreasing it for JA-Ile (−25%), IAA (−31%), and OPDA (−87%). These data proved that the components present in the matrix can indeed influence the response of each analyte in different ways and intensities. Therefore, one of the most reliable ways to evaluate all the matrix effects on quantitative results is using quantification methods (calibration curve and QC) fully developed in the presence of the matrix.

#### Repeatability, within-laboratory reproducibility, and inter-laboratory reproducibility

Repeatability, within-laboratory reproducibility and inter-laboratory reproducibility (Scheme 1) were evaluated in order to define the method's accuracy and precision (EMA and European Commission). They were assessed by overall mean, standard deviation and coefficient of variation for three QC levels (low, medium and high) for independent samples prepared using three spiking solutions.

Repeatability was evaluated by standard deviation and coefficient of variation of three batches (curve and QC) prepared in the same day by the same analyst. While the within-laboratory reproducibility was evaluated comparing the mean, standard deviation (RSD), and coefficient of variation (error) obtained during repeatability measurements and those obtained for samples prepared by a second analyst. The error and standard deviation for practically all QC (low, medium, and high) of all phytohormones were below 15% (Table 3). It indicates that this method is precise and accurate for quantification of phytohormone when the measurements are performed in a same laboratory (same equipment, solvents and standards) even when the samples are prepared by different analysts.

**Table 3:** **Values of repeatability, within-laboratory reproducibility and inter-laboratory reproducibility obtained during the validation of the method for quantification of various phytohormones (ABA, IAA, JA-Ile, SA, JA, and OPDA) in *Arabidopsis thaliana***.

	Expected conc. (ng/g FW)^*^	**Repeatability *n* = 3**			**Within laboratory reproducibility *n* = 6**			**Inter-laboratory reproducibility *n* = 9**		
		**Mean ± *SD***	**RSD (%)**	**Error (%)**	**Mean ± *SD***	**RSD (%)**	**Error (%)**	**Mean ± *SD***	**RSD (%)**	**Error (%)**
ABA	7.00	6.67 ± 0.59	8.91	−4.73	6.92 ± 0.56	8.11	−1.15	7.01 ± 0.31	4.37	0.21
	350.00	344.22 ± 11.30	3.28	−1.65	361.58 ± 26.76	7.40	3.31	359.20 ± 22.21	6.18	2.63
	1400.00	1331.25 ± 57.27	4.30	−4.91	1422.62 ± 144.62	10.17	1.58	1401.39 ± 122.85	8.77	0.10
IAA	7.00	7.12 ± 0.71	9.96	1.67	7.29 ± 0.47	6.43	4.09	7.36 ± 0.21	2.86	5.09
	350.00	313.07 ± 8.70	2.78	−10.55	326.16 ± 22.11	6.78	−6.81	332.73 ± 17.28	5.13	−4.94
	1400.00	1306.57 ± 63.12	4.83	−6.67	1378.70 ± 7.32	7.32	−1.52	1353.86 ± 105.62	7.80	−3.30
JA-Ile	1.40	1.57 ± 0.11	6.76	12.34	1.56 ± 0.10	6.36	11.76	1.54 ± 0.03	2.19	10.32
	70.00	70.30 ± 1.85	2.64	0.43	72.24 ± 4.02	5.56	3.20	71.04 ± 3.47	4.88	1.48
	280.00	273.76 ± 3.36	2.36	−2.23	290.30 ± 22.26	7.67	3.68	286.83 ± 22.10	7.71	2.44
SA	140.00	138.46 ± 16.48	11.9	−1.10	138.25 ± 11.59	8.38	−1.25	136.18 ± 3.54	2.60	−2.73
	1400.00	1367.10 ± 43.35	3.17	−2.35	1355.08 ± 84.25	6.22	−3.21	1321.77 ± 56.00	4.24	−5.59
	2800.00	2598.57 ± 112.39	4.32	−7.19	2667.01 ± 145.34	5.45	−4.75	2612.61 ± 139.18	5.33	−6.69
JA	70.00	73.62 ± 3.79	5.15	5.18	70.87 ± 5.63	7.95	−1.25	69.83 ± 3.36	4.82	−0.25
	700.00	691.744 ± 76.12	11.00	−1.18	690.70 ± 63.21	9.15	−1.33	674.38 ± 27.99	4.15	−3.66
	1400.00	1348.90 ± 59.33	4.40	−3.65	1371.20 ± 107.43	7.83	−2.06	1354.27 ± 44.22	3.27	−3.27
OPDA	225.00	220 ± 12.11	5.48	−1.78	233.74 ± 23.54	10.07	3.89	231.11 ± 17.02	7.36	2.71
	700.00	696.42 ± 105.51	15.15	−0.51	711.56 ± 76.76	10.79	1.65	685.69 ± 52.24	7.62	−2.04
	1400.00	1371.79 ± 187.81	13.69	−2.02	1450.72 ± 161.20	11.11	3.62	1411.55 ± 129.21	9.15	0.83

* Corresponds to ng/mL.

Inter-laboratory reproducibility was assessed by collaborative study with Accert Chemistry and Biotechnology Inc., where three new batches of both calibration curve and QC samples were prepared and analyzed using the same extraction procedure as described for repeatability. The error and standard deviation are very low (<10%). It proves that this method is precise and accurate and, hence, it can be used in different laboratories for quantification of phytohormones in *A. thaliana* tissues in order to generate directly comparable data. It is important to highlight that all measurements (calibration curve and QC) must be done in the matrix and for each batch of real samples a calibration curve and the QC (quintuplicate) must be analyzed before samples in order to guarantee the accurateness of the results.

#### Recovery

As described in Recovery, recovery was determined by the ratio between the amount of each phytohormone present in spiked/extracted and extracted/spiked samples. The extracted/spiked samples contained all the matrix interferes and 100% of the phytohormones concentration, since the standards were not subjected to the extraction procedure. On the other hand, in the spiked/extracted samples, the standards were added to the plant samples and the whole extraction procedure was performed afterwards. Thus, the spiked/extracted samples mimicked what happened with the phytohormones during the extraction procedure. The values of recovery for the different QC are shown in the Table 4. The overall recovery corresponds to the mean of recovery in different levels. For IAA and ABA the recovery was high, nearly 100%. However, for OPDA the overall recovery was 67.95%. It proved that the matrix affects the recovery distinctly depending on the analyte and on the concentration level. It also shows the significance of performing the calibration curve in the matrix and of validating the analytical method, once the different recoveries were enclosed for the entire range of the calibration curve developed in the matrix.

**Table 4:** **Percentage of recovery during the extraction of phytohormones in *Arabidopsis thaliana***.

**Compound**	**% of Recovery (Mean + Error)**			
	Low^*^	Medium^*^	High^*^	**Overall average of recovery**
IAA	88.94 ± 12.75	90.98 ± 15.64	97.09 ± 15.15	92.34 ± 4.24
ABA	98.60 ± 11.33	104.50 ± 7.48	105.48 ± 8.52	102.86 ± 3.72
JA-Ile	73.31 ± 13.18	80.99 ± 9.24	77.26 ± 8.50	77.19 ± 3.84
JA	85.35 ± 15.98	75.32 ± 5.71	75.65 ± 12.50	78.77 ± 5.70
SA	86.37 ± 9.31	86.72 ± 9.42	93.71 ± 9.39	88.93 ± 4.14
OPDA	80.05 ± 11.45	63.90 ± 10.97	59.89 ± 5.91	67.95 ± 15.70

* Corresponding to the concentrations given in Quality Controls.

#### Quantification of phytohormones in Citrus sinensis

In order to transfer this method to another plant, we choose one of the most the important fruit crops, *C. sinensis*. Thus, statistical parameters such as linearity, repeatability (accuracy and precision), matrix effect and recovery were also evaluated for quantification of phytohormones in leaves of this plant.

The basal level of each phytohormone is shown in Table 5. Both range of calibration curve and QC levels had to be modified in order to adjust the quantification method to the content of phytohormones present in citrus. As mentioned above it is important that the calibration curves include the concentration of the phytohormones present in the blank (untreated control) samples, since it usually corresponds to the control in biological experiments.

**Table 5:** **Parameters of calibration curve for each phytohormone: curve range, regression, weighting, correlation coefficient, limit of quantification (LOQ) and amount of each phytohormone present in blank *Citrus sinensis* samples**.

**Analyte**	**Range (ng/g FW)**	Curve^*^	***R*^2^**	**LOQ (ng/g)**	Amount in blank samples^**^ (ng/g FW)	Matrix effect^***^
IAA	25–4000	Y = 2.09382 + 0.0199062^*^X	0.989	25	111.47 ± 17.95	−16%
ABA	20–2000	Y = 3.463782 + 0.012662^*^X	0.994	20	262.07 ± 6.71	+2%
JA-Ile	0.4–400	Y = 0.176041 + 0.0702724^*^X	0.994	0.4	1.91 ± 0.10	+86%
JA	12.5–2000	Y = 0.74606 + 0.013142^*^X	0.987	12.5	54.32 ± 9.43	+147%
SA	25–4000	Y = 0.152181 + 0.00719421^*^X	0.998	25.0	29.60 ± 6.35	−4%
OPDA	30–600	Y = 1.41748 + 0.00743165^*^X	0.981	30	85.15 ± 1.49	−32%

* A weighting factor of 1/x^2^ was applied to all curves, except for OPDA, which used a factor of 1/x.

** Values are average ± standard deviation. Concentrations represent the amount of each phytohormone in plant tissues (ng/g of fresh weight, FW), which is corresponding to the concentration (ng/mL) in the injection solution.

*** Values correspond to ((m_matrix_/m_solvent_) −1)^*^100%.

Linear regression was used for all phytohormones calibration curves with weighting of 1/x^2^ for IAA, ABA, JA-Ile, JA, and SA and 1/x for OPDA. Linearity was assessed by correlation factor (Table 5) and matrix effect corresponds to the ratio between the angular coefficient of calibration curve in matrix and in solvent.

This particular matrix (*C. sinensis*) had a small effect in the calibration curve for IAA, ABA, and SA. However, for OPDA, JA-Ile, and JA the matrix had a strong influence in the inclination of the calibration curves (Table 5 and Figure D, Supplementary Material). The comparison between these results and those presented in Table 2 (for *A. thaliana*) highlights the importance of performing the calibration curve in the presence of each individual matrix, since they interfere differently in the quantification of each phytohormone.

Statistical parameters accuracy (error) and precision (RSD) were also evaluated for quantification of phytohormones in *C. sinensis* and the results are presented in Table 6. Basically, all values are lower than 15%, proving that this method is suitable for quantification of phytohormones in citrus.

**Table 6:** **Values of repeatability (accuracy and precision) obtained during the validation of the method for quantification of various phytohormones (ABA, IAA, JA-Ile, SA, JA, and OPDA) in leaves of *Citrus sinensis***.

	Expected conc. (ng/g FW)^*^	**Repeatability *n* = 3**			**% Recovery (mean + error)**	**Overall average of recovery**
		**Mean ± *SD***	**RSD (%)**	**Error (%)**		
ABA	70.00	73.13 ± 3.42	4.68	4.48	84.53 ± 13.04	82.77 ± 8.06
	350.00	369.09 ± 15.06	4.08	5.46	75.63 ± 8.08	
	1400.00	1479.93 ± 73.67	4.98	5.71	88.15 ± 4.83	
IAA	140.00	142.75 ± 9.55	6.69	1.97	55.65 ± 7.97	66.16 ± 8.51
	1400.00	1488.05 ± 23.62	1.59	6.29	70.29 ± 7.42	
	2800.00	3023.73 ± 164.79	5.45	7.99	72.54 ± 5.86	
JA-Ile	1.40	1.43 ± 0.17	11.87	2.38	39.74 ± 8.91	63.23 ± 10.90
	70.00	69.96 ± 2.74	3.92	−0.06	73.36 ± 7.59	
	280.00	300.15 ± 10.17	3.39	7.20	76.59 ± 5.82	
SA	140.00	160.94 ± 2.88	1.79	14.96	61.16 ± 5.56	73.95 ± 5.19
	1400.00	1611.94 ± 106.92	6.63	15.14	81.15 ± 7.58	
	2800.00	3205.07 ± 107.74	3.36	14.47	79.56 ± 3.75	
JA	70.00	76.17 ± 4.97	6.52	8.82	63.61 ± 8.97	75.59 ± 7.40
	700.00	747.05 ± 8.03	1.07	6.72	83.47 ± 7.73	
	1400.00	1496.15 ± 108.73	7.27	6.87	79.68 ± 4.81	
OPDA	90.00	90.67 ± 7.34	8.10	0.74	66.19 ± 10.58	71.00 ± 10.70
	300.00	303.39 ± 34.64	11.42	1.13	74.00 ± 9.77	
	420.00	450.81 ± 60.86	13.50	7.34	72.81 ± 6.57	

* Corresponding to the concentrations given in Validation in Citrus sinensis.

Recovery was calculated by comparison between spiked/extracted and extracted/spiking samples as described in Recovery. In the same way as discussed for matrix effects, recovery depends on both matrix and the nature of each compound. Recovery of IAA, for example, is strongly different between *Arabidopsis* and *Citrus*. Therefore, to compare the content of phytohormone in different matrix both calibration curve and recovery must be evaluated in every individual matrix.

### General comments

In the present work we developed and validated a reliable, precise and accurate method for quantification of six different phytohormones (IAA, ABA, SA, JA, JA-Ile, and OPDA) in tissues of two different plants, the model plant *A. thaliana* and the fruit crop *C. sinensis*. As it was possible to transpose the method to a second, independent laboratory, its applicability and reproducibility in different laboratory environments with different set-ups was successfully demonstrated. Moreover, we showed the significance of the validation of the analytical method for the understanding of analyte stability and the matrix effect in the different levels of the analyte concentrations and for different matrixes. This study shows that it is possible to reach comparable standards for phytohormone measurements, independent where the analyses are performed.

### Conflict of interest statement

The authors declare that the research was conducted in the absence of any commercial or financial relationships that could be construed as a potential conflict of interest.

## Supplementary material

Scheme 1 **Needs to establish a validated method**.
