# Supplementary figures and images for: Validated method for phytohormone quantification in plants
Source: Front Plant Sci. 2014 Aug 26;5:417. doi: 10.3389/fpls.2014.00417 (PMC4143963; doi:10.3389/fpls.2014.00417)

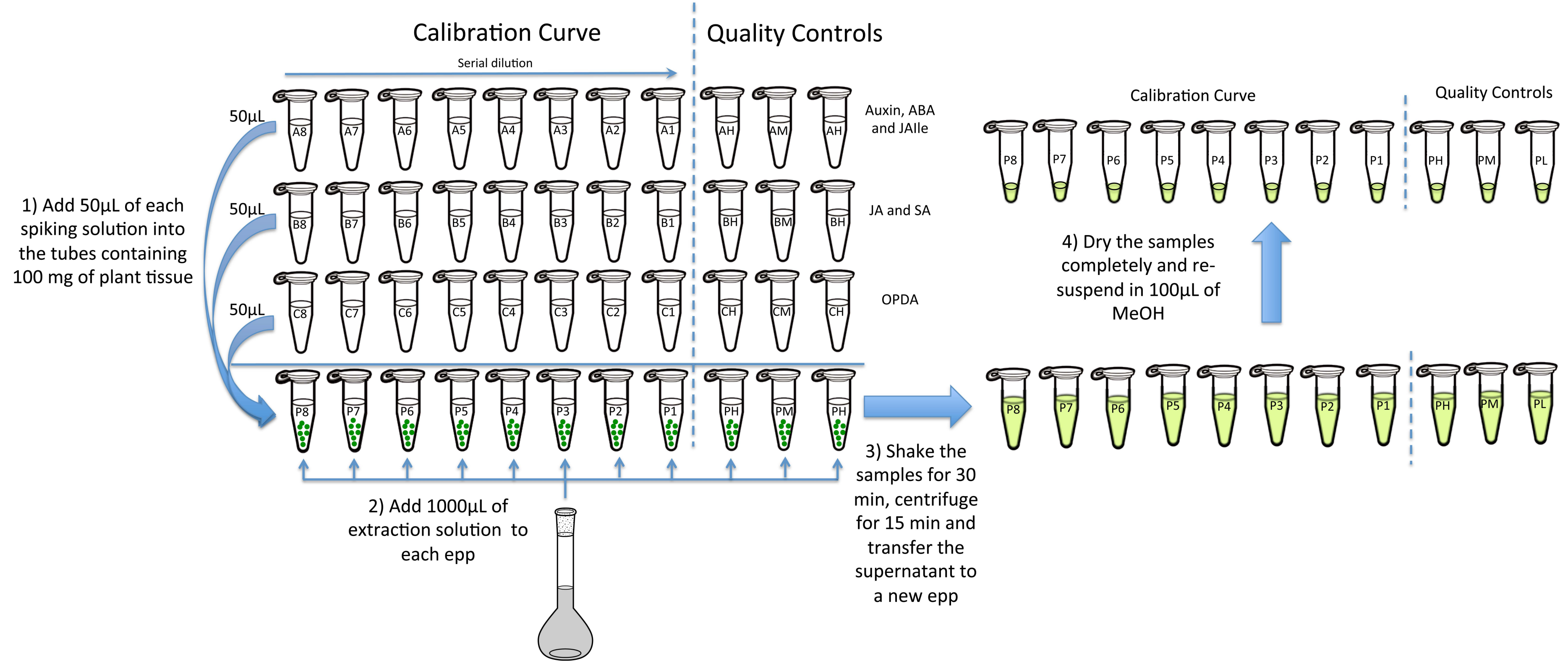

Supplement: Scheme 1 — Needs to establish a validated method. [file Presentation1.PDF]
