# Supplementary material for: Validated method for phytohormone quantification in plants
Source: Front Plant Sci. 2014 Aug 26;5:417. doi: 10.3389/fpls.2014.00417 (PMC4143963; doi:10.3389/fpls.2014.00417)

Supporting information

**Figure A**: Extraction of phytohormones according to different extraction solvents.


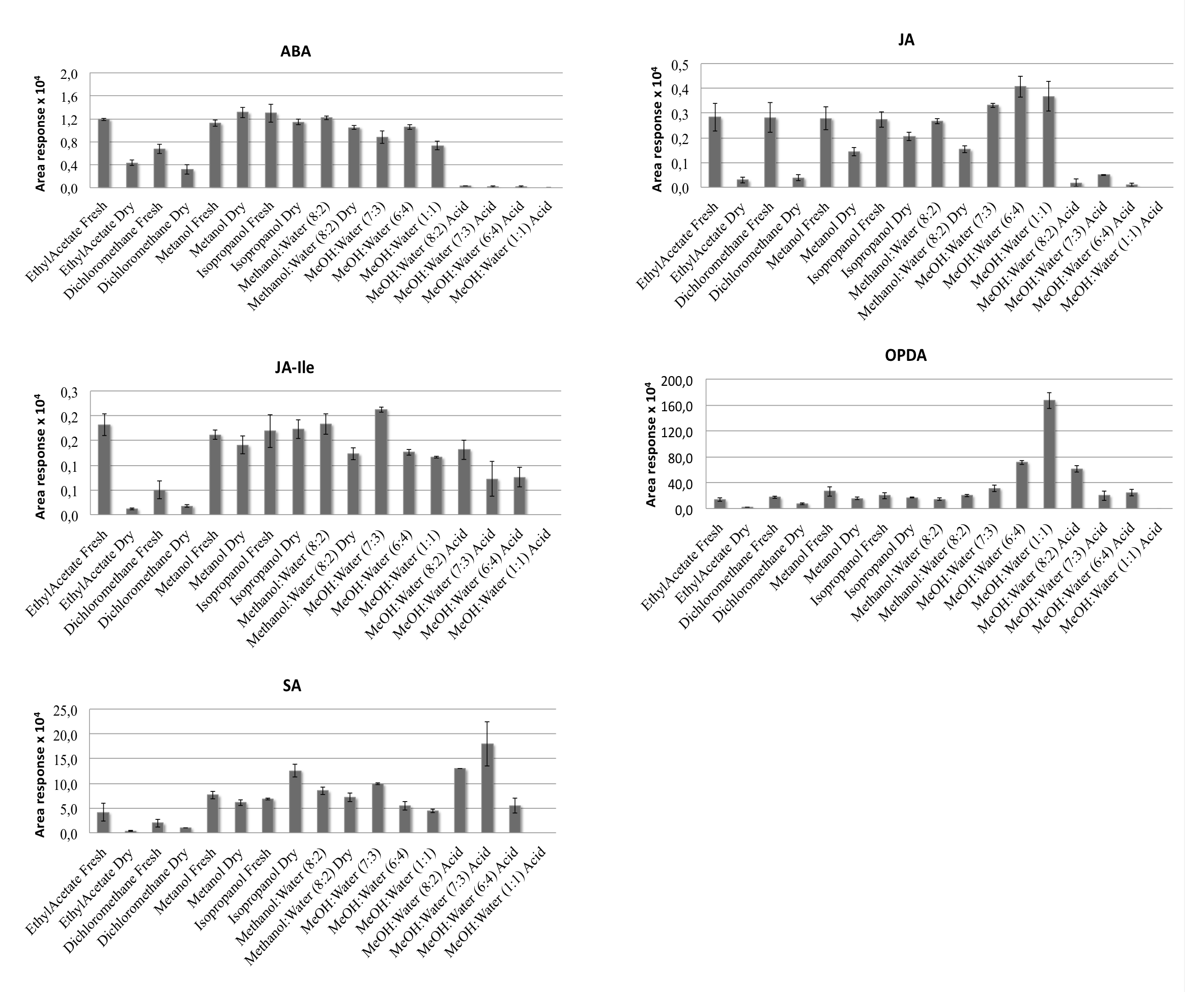


**Figure B**: Comparison between the MS/MS spectrum of original standards in solvent and in the matrix.


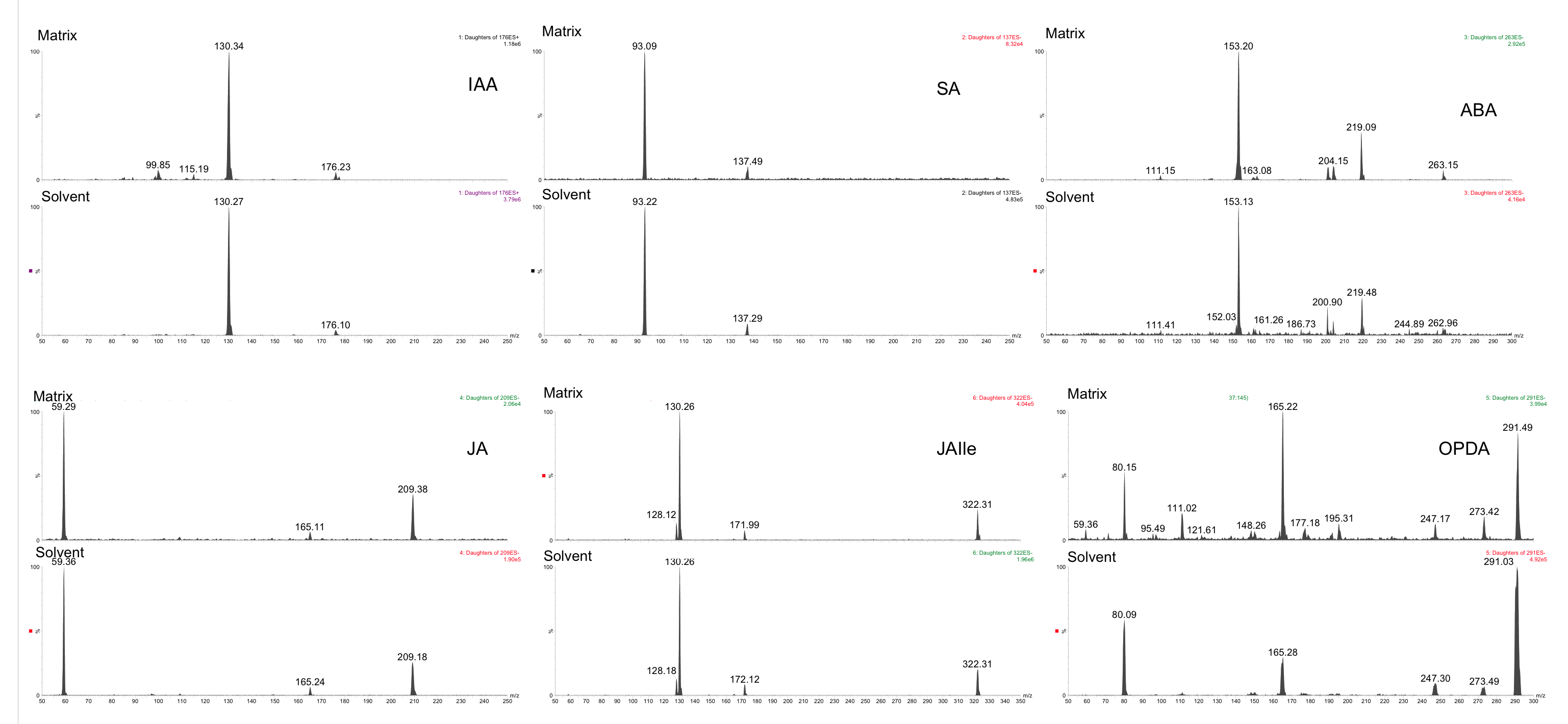


**Figure C**: SRM chromatograms of the lowest points of calibration curve for each phytohormone. (IAA:, SA:, ABA:, JA:, JAIle:, and OPDA:


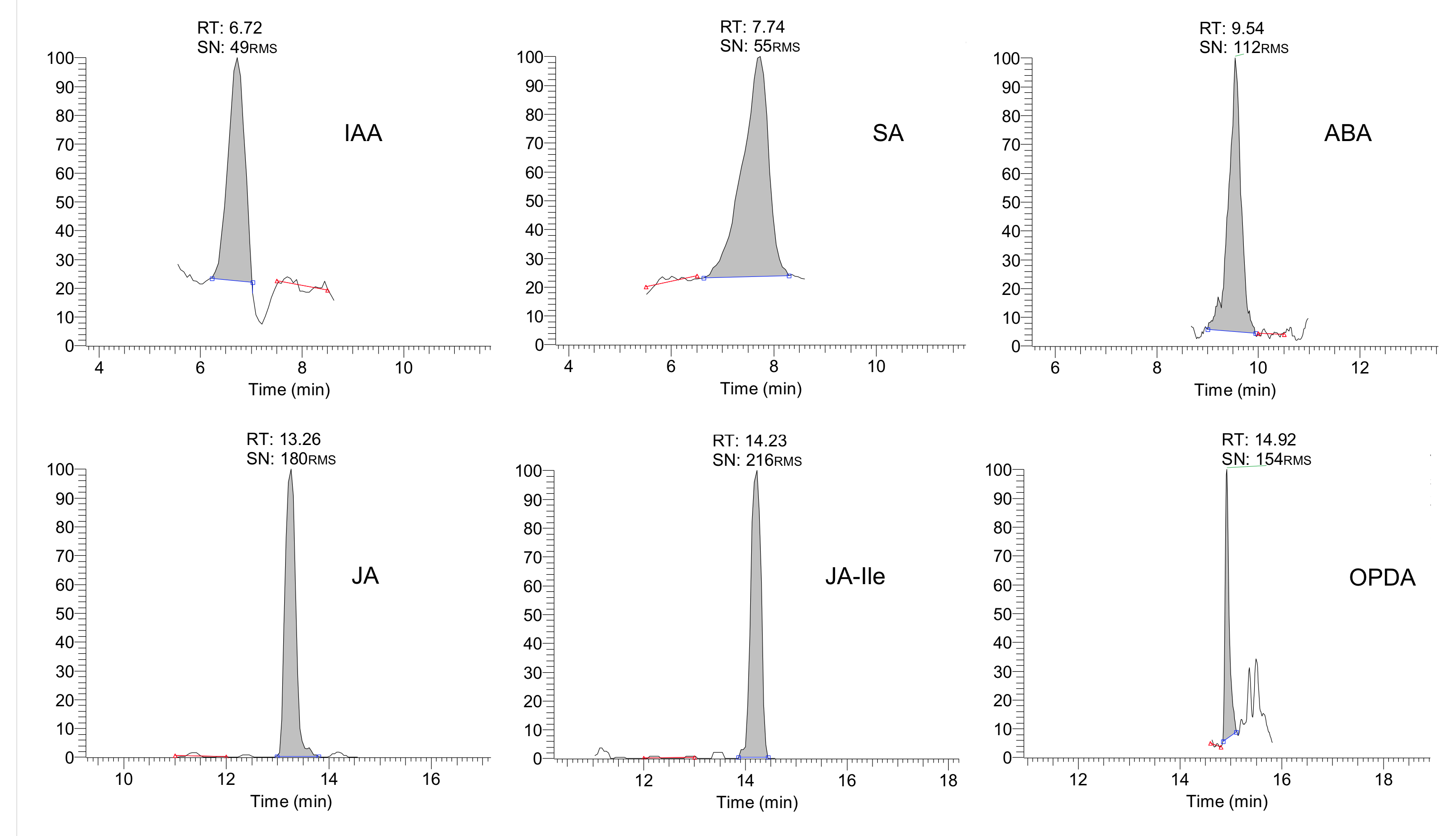


**Figure D**: Comparison between calibration curves performed in the matrix (*Citrus sinensis)* and in solvent.


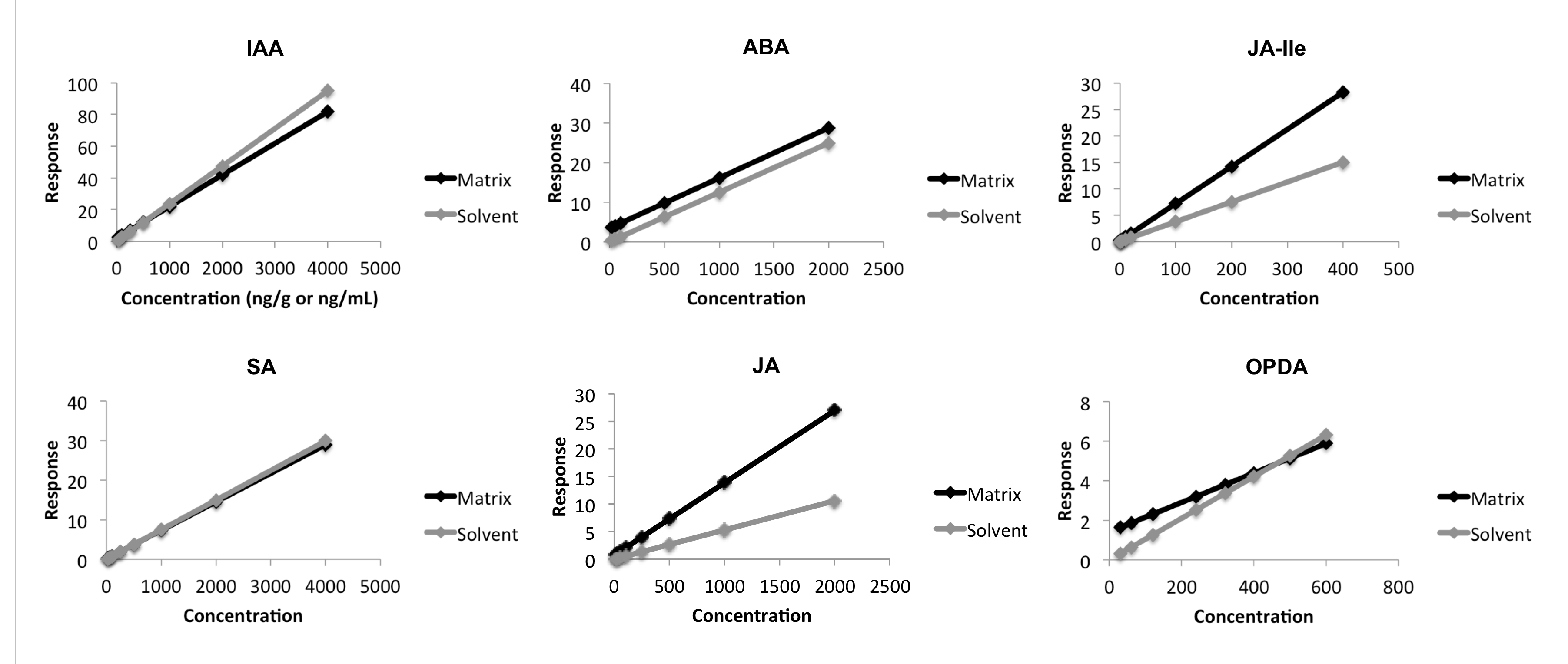

Supplement: Supplementary file 2 [file DataSheet1.DOCX]
